# Molecular characterization of metastatic exon 11 mutant gastrointestinal stromal tumors (GIST) beyond KIT/PDGFRα genotype evaluated by next generation sequencing (NGS)

**DOI:** 10.18632/oncotarget.6278

**Published:** 2015-11-02

**Authors:** Maristella Saponara, Milena Urbini, Annalisa Astolfi, Valentina Indio, Giorgio Ercolani, Massimo Del Gaudio, Donatella Santini, Maria Giulia Pirini, Michelangelo Fiorentino, Margherita Nannini, Cristian Lolli, Anna Mandrioli, Lidia Gatto, Giovanni Brandi, Guido Biasco, Antonio Daniele Pinna, Maria Abbondanza Pantaleo

**Affiliations:** ^1^ Department of Specialized, Experimental, and Diagnostic Medicine, Sant'Orsola-Malpighi Hospital, University of Bologna, Bologna, Italy; ^2^ Interdepartmental Centre of Cancer Research “G. Prodi”, University of Bologna, Bologna, Italy; ^3^ Department of General and Emergency Surgery and Organ Transplantation, S. Orsola-Malpighi Hospital, University of Bologna, Bologna, Italy; ^4^ Pathology Unit, S. Orsola-Malpighi Hospital, University of Bologna, Bologna, Italy; ^5^ Laboratory of Molecular Oncologic and Transplantation Pathology, S. Orsola-Malpighi Hospital, University of Bologna, Bologna, Italy

**Keywords:** GIST, CCND2, NGS, PTEN, CDKN2C, DMD

## Abstract

About 85% of GISTs are associated with KIT and PDGFRα gene mutations, which predict response to tyrosine kinase inhibitors. Although the outcomes in patients affected by GIST have dramatically improved, tumor progression control still remains a challenge. The aim of this study is the genomic characterization of individual metastatic KIT-exon 11-mutant GIST to identify additional aberrations and simultaneous molecular events representing potential therapeutic targets.

Seven patients with metastatic GIST were studied with whole transcriptome sequencing and copy number analysis. Somatic single nucleotide variations were called; however, no shared mutated genes were detected except KIT. Almost all patients showed loss of genomic regions containing tumor suppressor genes, sometimes coupled with single nucleotide mutation of the other allele. Additionally, six fusion transcripts were found and three patients showed amplifications involving known oncogenes.

Evaluating the concordance between CN status and mRNA expression levels, we detected overexpression of CCND2 and EGFR and silencing of CDKN2A, CDKN2C, SMARCB1, PTEN and DMD. Altered expression of these genes could be responsible for aberrant activation of signaling pathways that support tumor growth. In this work, we assessed the effect of Hedgehog pathway inhibition in GIST882 cells, which causes decrement of cell viability associated with reduction of KIT expression.

Additional genomic alterations not previously reported in GIST were found even if not shared by all samples. This contributes to a more detailed molecular understanding of this disease, useful for identification of new targets and novel therapeutics and representing a possible point of departure for a truly individualized clinical approach.

## INTRODUCTION

Gastrointestinal stromal tumors (GISTs) are the most common mesenchymal tumors of the gastrointestinal tract. About 90% of GISTs are associated with KIT or PDGFRα gene mutations. The remaining 10-15% of GISTs do not harbor identified receptor tyrosine-kinase mutations (KIT/PDGFRα wild-type GIST) and are very different from mutant GISTs in their clinical and molecular profiles, to the extent that they are now considered a separate pathological entity with wide heterogeneity [1, 2].

The in-depth understanding of GIST pathogenesis led to the development of molecular targeted therapies with tyrosine kinase inhibitors (TKIs). KIT and PDGFRα mutational status predicts clinical response to imatinib and therefore molecular sub-classification of GISTs is essential to recognize patients who will benefit from therapy [3-4].

Unfortunately, even imatinib-responsive patients quite invariably acquire resistance after a median time of about 24 months [5]. In general, the mechanisms of secondary resistance are divided between those associated with KIT/PDGFRα receptors, among which secondary mutations are believed to be most prevalent and frequent, and those mechanisms independent from KIT/PDGFRα receptors, such as (i) chromosomal alterations (such as loss of chromosomes 1p, 14q or 22q), (ii) pharmacokinetic variables, and (iii) tumor differentiation (such as sarcomatoid differentiation).

Secondary mutations have been found mainly in patients who initially had primary KIT mutations and rarely in those with primary PDGFRα mutations. Secondary mutations were most often found in the ATP-binding pocket (exon 13 and 14) or in the kinase activation loop (exon 17 and 18), producing alterations in KIT conformation and thus decreasing imatinib affinity [6]. Instead no secondary KIT/PDGFRα mutations have been documented in KIT/PDGFRα wild-type GIST tumors, suggesting that imatinib binding could be providing selective pressure.

To treat patients with acquired imatinib resistance, a second generation of TKIs, including sunitinib and regorafenib, has been developed; these TKIs also target the kinases involved in tumor-related angiogenesis [7-9]. The need of prolonging life expectancy of patients associated with the complex biology of progressive disease has led to a growing urgency and interest in the understanding the basic biology of GISTs and in the identification of new strategies to overcome resistance, including new molecules, drug-drug combinations, and the integration of loco-regional treatments [10-13].

Over the last decade, advances in next generation sequencing (NGS) technology enabled simultaneous examination of numerous gene analyses. The rationale behind the quest for additional genetic events grows out of the heterogeneity of clinical outcomes and GIST patients' treatment responses, which suggests that molecular events other than gain-of-function mutations in KIT and PDGFRα are involved in the biological behavior of tumor aggressiveness.

The aim of this study is a genome analysis for the characterization of individual metastatic KIT exon 11-mutant GIST, to better understand the range of additional aberrations occurring in each tumor and eventually find simultaneous molecular events that are responsible for tumor progression and may represent potential new drug target candidates.

## RESULTS

### SNV, INDEL and fusion detection

Seven patients with metastatic GIST harboring a KIT exon 11 mutation were studied (Table 1). For each case, WTS was performed on RNA isolated from fresh-frozen tissue and WES was done on DNA isolated from matched peripheral blood.

**Table 1:** Clinical and molecular patient characteristics

Patient ID	Sex	Age	Site	Distant metastasis	Current status	KIT mutational status
GIST_11	M	77	Stomach	Peritoneum	NED (surgical patient)	KIT exon 11 c.1669_1673del
GIST_124	M	73	Stomach	Peritoneum Lung	DOD	KIT exon 11 c.1765-1766 ins
GIST_131	M	68	Ileum	Liver	AWD	KIT exon 11 c.1706_1735del
GIST_178	F	70	Stomach	Liver Peritoneum	AWD	KIT exon 11 c.1676T>A
GIST_150	F	58	Stomach	Liver	NED (surgical patient)	KIT exon 11 c.1651_1663del
GIST_174	M	61	Stomach	Liver	NED (surgical patient)	KIT exon 11 c.1727T>C
GIST_188	F	62	Duodenum	Liver Bone	DOD	KIT exon 11 c.1690_1728del39 KIT exon 17 c.2466T>G

NED: no evidence of disease; DOD: dead of disease; AWD: alive with disease

RNA libraries were sequenced to an average of 42-fold of tumor coverage. In order to identify novel single nucleotide variations (SNVs), all variants found in the dbSNP131 or 1000 Genomes Project with MAF greater than 0.01 were excluded. An average of 172 (range, 151-195) novel SNVs were found in each tumor sample, of which an average of 11,4 (range, 7-17) were somatic. The effect of these putative mutations were predicted using two bioinformatic tools: SNPs&GO and PROVEAN. The list of all novel somatic SNVs and insertions and deletions (INDELs) predicted to be deleterious by at least one tool is provided in Table 2.

**Table 2:** All novel somatic SNVs and INDELs detected in metastatic KIT-mutated GISTs and predicted deleterious with at least one predictor

GENE	exon	cDNA mutation	Protein mutation	Alteration type	Chr.	Genomic coordinate	Reference Base	Altered Base	Coverage Ratio Tumor RNA seq	SNPs&GO prediction	PROVEAN prediction
**GIST_11**											
**KIT**	**11**	c.1669_1673del	p.557_558del	Non frameshift deletion	4	55593601	AGTGGA	-	0.43	NA	Deleterious
DDT	2	c.G280A	p.D94N	SNV	22	24315961	C	T	0.72	Deleterious	Deleterious
GNAI3	3	c.G299C	p.R100T	SNV	1	110116655	G	C	0.34	Neutral	Deleterious
**GIST_124**											
**KIT**	**11**	c.1765-1766ins	p.589insQLPYDHWEFPRNR	Non frameshift insertion	4	55593601	-	CAACTATTCTATGACCACTGGGAATTTCCTAGGAACAGG	-	NA	Deleterious
CDKN2A	2	c.C247T	p.H83Y	SNV	9	21971111	G	A	1.00	Deleterious	Deleterious
FAM21C	9	c.T746C	p.M249T	SNV	10	46245557	T	C	0.52	Neutral	Deleterious
PCTP	2	c.G188A	p.C63Y	SNV	17	53844742	G	A	0.59	Deleterious	Deleterious
TRIM28	4	c.T658C	p.F220L	SNV	19	59058814	T	C	0.38	Neutral	Deleterious
UBR1	32	c.A3614G	p.N1205S	SNV	15	43294798	T	C	0.59	Neutral	Deleterious
AUP1	2	c.75delG	p.L25fs	Frameshift deletion	2	74756602	C	-	0.54	NA	frameshift
**GIST_131**											
**KIT**	**11**	c.1706_1735del	p.569_578del	Non frameshift deletion	4	55593638	TGTTTACATAGACCCAACACAACT	-	0.52	NA	Deleterious
ZCCHC11	26	c.A3965C	p.K1321T	SNV	1	52902624	T	G	0.96	Neutral	Deleterious
ALDOB	5	c.C524A	p.A175D	SNV	9	104189780	G	T	0.55	Deleterious	Deleterious
NRP1	5	c.G800A	p.S267N	SNV	10	33545258	C	T	0.45	Deleterious	Neutral
POTEE	15	c.2387_2409del	p.796_803del	Frameshift deletion	2	132021415	TGGCTCCCGAGGAGCACCCCATC	-	0.55	NA	frameshift
**GIST_150**											
**KIT**	**11**	c.1651_1663del	p.551_554del	Non frameshift deletion	4	55593583	AACCCATGTATG	-	0.99	NA	Deleterious
ASUN	9	c.G923C	p.G308A	SNV	12	27070633	C	G	0.60	Neutral	Deleterious
KEAP1	4	c.G1385A	p.G462E	SNV	19	10600470	C	T	0.42	Deleterious	Deleterious
KIAA0564	16	c.C1874T	p.P625L	SNV	13	42390907	G	A	0.50	Neutral	Deleterious
TMEM8A	4	c.G509C	p.G170A	SNV	16	427163	C	G	0.36	Neutral	Deleterious
FNBP4	10	c.1607_1608 ins	p.T536fs	Frameshift insertion	11	47755656	-	T	0.94	NA	frameshift
CSAD	8	c.745delC	p.L249fs	Frameshift deletion	12	53552333	G	-	0.60	NA	frameshift
**GIST_174**											
**KIT**	**11**	c.T1727C	p.L576P	SNV	4	55593661	T	C	1.00	Deleterious	Deleterious
LATS2	8	c.C2809T	p.Q937X	Stop gained	13	21549467	G	A	0.40	Deleterious	Deleterious
PRPF31	12	c.G1153C	p.E385Q	SNV	19	54632438	G	C	0.47	Neutral	Deleterious
PTEN	7	c.C697T	p.R233X	Stop gained	10	89717672	C	T	0.69	Deleterious	Deleterious
TOMM70A	2	c.T357A	p.N119K	SNV	3	100105790	A	T	0.91	Neutral	Deleterious
ZNF12	6	c.C1864T	p.P660S	SNV	7	6730595	G	A	0.71	Neutral	Deleterious
**GIST_178**											
**KIT**	**11**	c.T1676A	p.V559D	SNV	4	55593610	T	A	0.63	Deleterious	Deleterious
CES1	7	c.C852G	p.H284Q	SNV	16	55853498	G	C	1.00	Neutral	Deleterious
SGSM2	11	c.A1247G	p.Y416C	SNV	17	2268594	A	G	0.32	Neutral	Deleterious
IL17RC	19	c.1835_1839del	p.612_613del	Frameshift deletion	3	9974736	CGCTG	-	0.68	NA	frameshift
**GIST_188**											
**KIT**	**11**	c.1690_1728del	p.564_576del	Non frameshift deletion	4	55593609	AATGGAAACAATTA.....CATAGACCCAACACAACTT	-	0.51	NA	Deleterious
**KIT**	17	c.T2466G	p.N822K	SNV	4	55599340	T	G	0.46	Deleterious	Deleterious
EAF1	2	c.T155C	p.V52A	SNV	3	15471471	T	C	0.54	Neutral	Deleterious
HSPA1L	2	c.C1472T	p.T491I	SNV	6	31778278	G	A	0.64	Neutral	Deleterious
SHC1	5	c.C368T	p.T233I	SNV	1	154941023	G	A	0.42	Neutral	Deleterious
TAPT1	14	c.G1600T	p.E534X	Stop gained	4	16165035	C	A	0.59	Deleterious	Deleterious
TEF	4	c.G758A	p.R283H	SNV	22	41791900	G	A	0.97	Deleterious	Deleterious
CDKN1B	2	c.479delC	p.S160fs	Frameshift deletion	12	12871762	C	-	0.85	NA	frameshift
EIF4G3	9	c.1277_1279del	p.426_427del	Non frameshift deletion	1	21268200	GAG	-	0.97	NA	Deleterious
TIMM22	4	c.568_569ins	p.D190insDY	Non frameshift insertion	17	904311	-	ATT	0.26	NA	Deleterious

NA=analysis not available for insertion or deletion

The WTS analysis did not detect any shared mutated genes among the patients other than KIT, which was found mutated in six of the seven patients. The insertion mutation in patient GIST_124 was not detected by NGS analysis due to the length of the insertion fragment (39 bp), so the mutational status of this patient was assessed by Sanger sequencing only.

No known oncogenes were found mutated, but four of the seven patients (GIST_150, _174, _124 and _188) showed deleterious mutations in two candidates (KEAP1 and LATS2) and in three well-known tumor suppressors (CDKN2A, CDKN1B and PTEN).

WTS analysis revealed also the presence of six fusion transcripts (Table 3 and Figure S1), of which only the two in patient GIST_150 retained the reading frame. While the predicted chimeric protein DOK6-MYO5B seems to contain no functionally active domain of the two proteins involved, the predicted MEAF6-SEPSECS fusion protein retains the histone acetyltransferase subunit NuA4 domain of MEAF6. Additionally the presence of this last rearrangement was supported by the detection of copy number alteration in this region (Figure S2).

**Table 3:** Fusion events detected with RNA-sequencing in three metastatic KIT-mutated GISTs

Sample	5′ gene	Chr 5′ gene	5′ breakpoint mRNA exon	3′ gene	Chr 3′ gene	3′ breakpoint mRNA exon	N° SPLIT READS	N° SPAN READS	Frame retained	Fusion Event
**GIST_150**	DOK6	chr18	ex3	MYO5B	chr18	ex34	34	9	YES	Intra-chromosomal Complex
**GIST_150**	MEAF6	chr1	ex4	SEPSECS	chr4	ex8	21	6	YES	Inter-chromosomal
**GIST_178**	PACS2	chr14	ex1	IGHD	chr14	ex1	25	8	no	Intra-chromosomal Complex
**GIST_178**	FAM174A	chr5	ex1	DMGDH	chr5	ex16	26	16	no	Intra-chromosomal Complex
**GIST_131**	KDM4B	chr19	ex2(not coding)	ODF3L2	chr19	ex3	17	19	no	Intra-chromosomal Complex
**GIST_131**	KDM4B	chr19	ex2(not coding)	SHC2	chr19	ex2	12	5	no	Intra-chromosomal Complex

### Integration with copy number variation analysis

**Figure 1:**
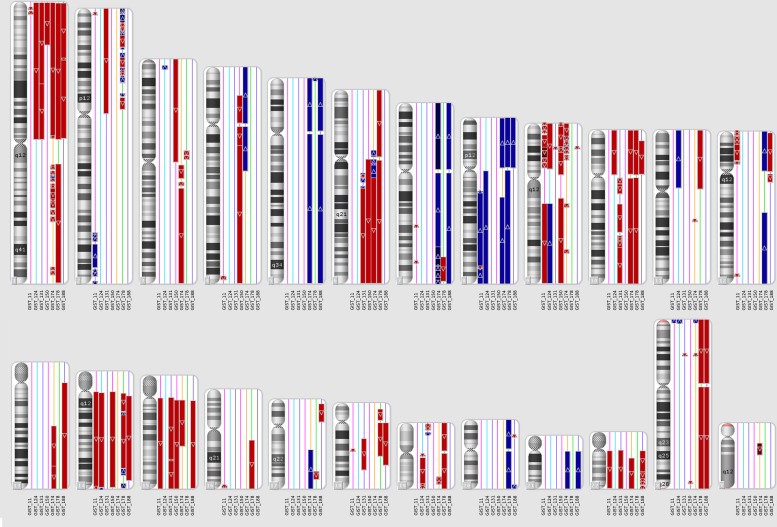
CNV analysis on metastatic KIT-mutated GIST Genomic region losses (red) or gains (blue) are shown for each chromosome in each patient. GIST178 had a triploid genome, so only in this case the CN=3 state was not considered altered, while regions with CN=2 were considered as LOSS of one allele and CN=4 were considered as GAIN of one copy.

SNP-array identified focal and macroscopic amplifications or deletions in all metastatic KIT-mutated GISTs. All patients showed at least two of the three common events of chromosome arm loss reported in GIST: 1p, 14q or 22q (Figure 1).

The analyses were then restricted to genomic events that affected target “driver” genes, thus it was focused on focal gains with copy number (CN) higher than 3, likely involving dominant oncogenes, or on focal losses affecting known tumor suppressors or homozygous losses (selected using the Cancer Genes Census database http://cancer.sanger.ac.uk/cosmic/census).

**Figure 2:**
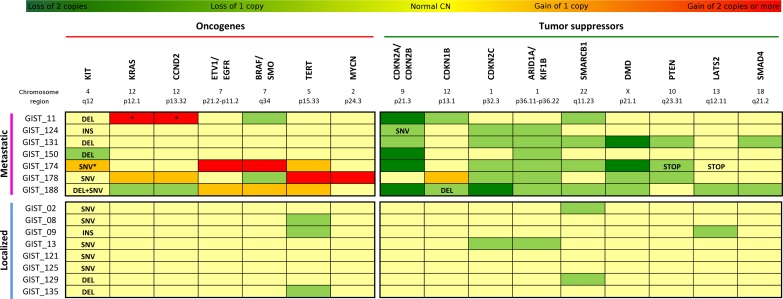
Mutational profile of KIT-mutated GIST Seven cases of metastatic KIT-mutated GIST are analyzed by RNA-seq and SNP-array, and data obtained are merged in the upper panel of the figure. Comparison with SNP-array data of eight patients with localized GIST is shown in the lower panel. In dark green are represented the homozygous loss, in light green the loss of one copy. Similarly in orange are the genes carrying gains of one copy, and in red the genes with gains of two or more copies. Light yellow indicates that the genes have a normal CN state. For each patient are reported the major oncogenes (left panel) and the major tumor suppressors (right panel) altered. INS=insertion; DEL=deletion; SNV=single nucleotide variation; STOP=stop codon gained. *Loss of heterozygosity. §GIST178 had a triploid genome.

Merging the data obtained from WTS and CNV analyses, it was possible to complete the mutational profile of oncogenes and tumor suppressors in the metastatic KIT-mutated GIST group (Suppl. Table 1). Recurrence of copy alterations in these genes was evaluated also in eight patients with localized KIT-mutated GIST (Figure 2). In addition to the first mutation on exon 11, a secondary alteration on KIT was detected in three patients with metastatic disease. While patient GIST_188 showed a second mutation on exon 17, GIST_150 and GIST_174 expressed only the mutated KIT; in the first case it was due to an LOH event, while in the other it was due to an amplification (CN = 3) of the mutated allele. No localized tumor carried secondary mutation of KIT.

Among the patients in the metastatic group, amplifications involving known oncogenes were detected in three of the seven tumors, while no alterations of these genes were detected in the localized tumors. In specific, patient GIST_11 carried a focal amplification of CCND2 (CN = 6) and KRAS (CN = 4); GIST_174 was affected by amplification of BRAF (CN = 6), ETV1 and EGFR (CN = 4); and GIST_178 showed remarkable focal amplifications involving MYCN (CN = 5) and TERT (CN = 6) genes.

Similarly, almost all metastatic patients showed loss of genomic regions containing tumor suppressor genes, and few events were detected in the localized group. CDKN2A and CDKN2B losses were the most frequent event; in particular, chr9 p21.3 region was deleted in homozygosis in four patients (GIST_11, _150, _174, _188) and in heterozygosis in two patients (GIST_124 and_131). As described above, in addition to the heterozygous deletion of this locus, patient GIST_124 carried a deleterious SNV in the CDKN2A gene that led to the complete loss of function of the protein. It is noteworthy that none of the localized tumors carried alterations of CDKN2A/B. Furthermore, two other cyclin-dependent kinase inhibitors were altered. In particular, one allele of CDKN2C (chr1) was deleted in five patients, and both alleles were lost in patient GIST_188. This latter patient also lost one copy of chr12p arm, coupled with a frameshift deletion in the CDKN1B gene. One copy loss of CDKN2C was also detected in one localized tumor.

The loss of the chromosome arm 1p in six patients with metastatic disease (GIST_124, _131,_178,_150, _174, _188) led also to one copy deletion of KIF1B and of the tumor suppressor ARID1A, a known SWI/SNF chromatin remodeling gene. Another member of this family, SMARCB1 on the chromosome arm 22q, was deleted in four patients (focal in GIST_188, and whole arm in GIST_11, _131, _174). Loss of one copy of ARID1A and SMARCB1 was detected also in one and two localized tumors respectively. Moreover, the loss of PTEN was detected in patients GIST_131 and GIST_174 (in this latter patient, the remaining allele carried a premature STOP codon) and one copy focal loss of SMAD4 was detected in GIST_131 and _188.

Finally, patient GIST_174 harbored two focal homozygous deletions in two putative tumor suppressors: COP1 on chr1 (a protein that negatively regulates ETV1, ETV4 and ETV5) and DMD on chr X. This last gene was also focally lost in patient GIST_131, and one copy loss was detected in GIST_178 and GIST_188.

### Gene expression of candidate genes

**Figure 3:**
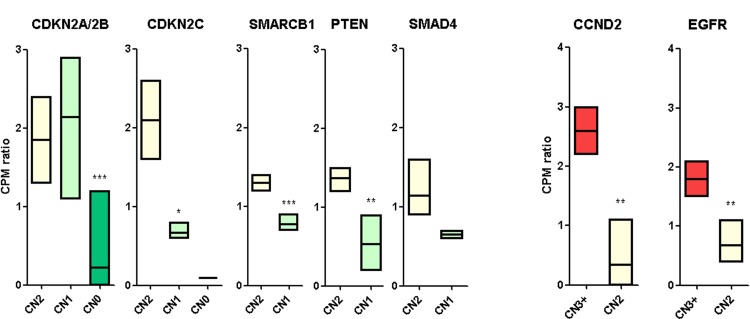
mRNA expression of candidate cancer-related genes mRNA expression level of cancer related genes are shown according to their CN status. Tumor suppressor genes are shown in the left panel, oncogenes in the right. Gene expression levels were evaluated using the cpm (normalized count of reads mapping each gene) normalized on the mean cpm of the cohort. The *p* value of differential expression was estimated with a t test: *p < 0,05; **p < 0,01; ***p < 0,001.

The expression levels of the cancer-related genes identified above were determined from RNA-seq data, with the aim to find the concordance between copy number status and mRNA expression (Figure 3). Interestingly, among the oncogenes, CCND2 and EGFR perfectly correlated between the two analysis performed, suggesting that the oncogenic events in these two chromosomal regions (12p13 and 7p12 respectively) could be the amplification and the resulting overexpression of these two genes. In particular, patients GIST_11 (CN = 6) and GIST_178 (CN = 3) showed a three-fold amplification of CCND2 with respect to disomic samples (*p* = 0,0027). The same was detected for EGFR in patients GIST_174 (CN = 4) and GIST_188 (CN = 3), with an average of two-fold overexpression (*p* = 0,0066). The other oncogenes did not showed an evident correlation between CN status and mRNA expression. For example, KRAS was also overexpressed in CN = 2 samples (GIST_150 and _174) in addition to GIST_11; (CN = 4) (data not shown). On the other hand, among the tumor suppressor genes, CDKN2A/2B showed a highly significant silencing of the two mRNAs in the homozygously deleted samples (GIST_11, _150, _174 and _188) with respect to both CN = 2 and CN = 1 samples. The simultaneous presence of a CN loss and a SNV of CDKN2A/2B in patient GIST_124 did not affect the mRNA expression level. Conversely, the other tumor suppressor genes showed significant down-regulation also in samples with CN = 1 status. In particular, CDKN2C mRNA, located in 1p32 chromosome region, was significantly underexpressed in CN = 1 samples (GIST_124, _131, _174 and _178) and there was almost no CDKN2C mRNA expression detected in patient GIST_188 (CN = 0). Moreover PTEN (*p* = 0,0062), SMARCB1 (*p* = 0,0009) and SMAD4 (not significant) showed a marked gene expression reduction in concordance with CN status. Finally, DMD deletions in patients GIST_131, _174 and _188 were also associated with a significant down-modulation of mRNA expression, as reported elsewhere [14].

### Hedgehog pathway inhibition

**Figure 4:**
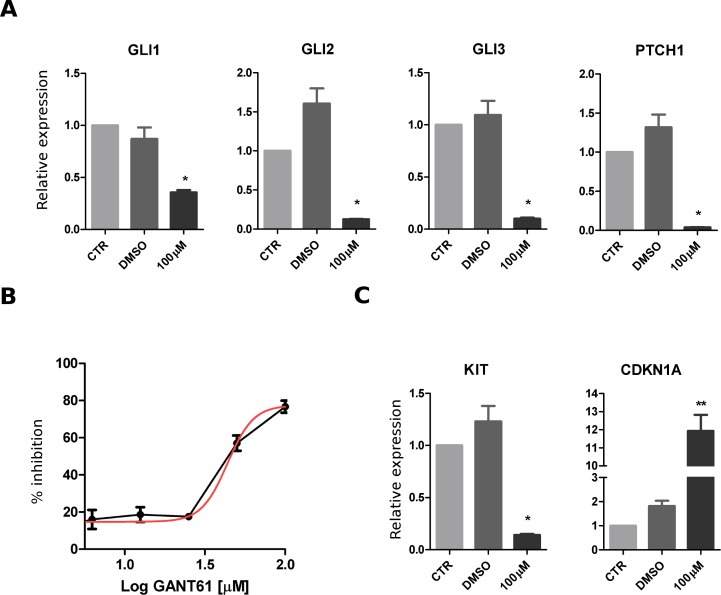
Effect of Hedgehog pathway inhibition in GIST882 cell line GIST882 cells were treated with GANT61 (concentration range, 6.25-100 μM) for 72 hours. **A.** The mRNA relative expression of hedgehog pathway genes (GLI1, GLI2, GLI3, PTCH1) was significantly downregulated in treated samples. **B.** Decrease of cell viability shown after GANT61 treatment. **C.** Effect of GANT61 on mRNA expression with downregulation of KIT and upregulation of CDKN1A. The p value of differential expression with respect to vehicle control (DMSO-only) was estimated with a t test: *p < 0.05; **p < 0.01.

GIST882 cells were exposed to GANT61, a GLI small-molecules agonist, to assess the effect of Hedgehog pathway inhibition. To assess the specificity of GANT61 treatment, relative quantification of Hedgehog pathway gene expression was evaluated by real-time PCR. mRNA levels of GLI1, GLI2, GLI3, PTCH1 were significantly down-regulated under treatment condition (Figure 4A). After 72 hrs treatment with scalar doses of GANT61, the calculated dose response curve showed an important inhibition of cell viability (Figure 4B). This effect was accompanied by a significant down-regulation of KIT and by up-regulation of CDKN1A mRNA expression (Figure 4C).

## DISCUSSION

In our study, we performed a WTS analysis on seven patients with metastatic KIT exon 11-mutant GIST. Analysis of fusion transcripts did not reveal any recurrent events. However, the MEAF6-SEPSEC rearrangement detected in one patient, GIST_150, is noteworthy since MEAF6 has recently been reported as translocated with PHF1 in endometrial stromal sarcomas and in ossifying fibromyxoid tumors [15-17]. Conversely, integration of WTS with CNV analysis allowed the identification of a mutational profile specific for metastatic KIT exon11-mutated GIST. In addition to the first mutation, a secondary alteration on KIT was detected in three patients with metastatic disease and in none of the patients with localized tumors. Moreover, several tumor suppressors (CDKN2A/B, CDKN1B, CDKN2C, ARID1A, KIF1B, SMARCB1, PTEN, DMD, LATS2 and SMAD4) and oncogenes (CCND2, KRAS, BRAF, ETV1, EGFR, MYCN, SMO and TERT) were found recurrently altered in metastatic GIST specimens and rarely in localized tumors. In particular, CDKN2A/B, PTEN and DMD were found altered exclusively in the metastatic cohort. With the aim of identifying potential pathogenic driver events and evaluating the possible effects of these alterations, the concordance between CN status and mRNA expression level was assessed.

Among oncogenes, the most significant overexpressed genes detected in association with copy number gains are CCND2 and EGFR, found respectively in the 12p13 and 7p12 chromosomal regions.

CCND2 is a well-conserved cyclin that forms a complex with CDK4 or CDK6, whose activity is required for cell cycle G1/S transition. High-level expression of this gene was observed in ovarian and testicular tumors [18, 19]. In our cohort CCND2 was found amplified and overexpressed in two metastatic samples.

Mutations of EGFR lead to ligand overexpression, which has been associated with a number of cancers. In consequence, the identification of EGFR as an oncogene has led to the development of many therapeutic approaches aimed at the EGFR pathway [20-23]. In our study two advanced GISTs showed amplification and high expression of EGFR, but EGFR overexpression should be evaluated in studies in larger cohorts to assess the possibility of targeting this pathway to treat GISTs.

With CNV analysis, we also found amplification of two known oncogenes, KRAS and BRAF. While somatic activating mutations of KRAS and BRAF proteins are common in several types of malignancies, including GISTs [24-30], and they may correlate with response to the specific inhibitors [31], few data are available on their overexpression [32-35]. Our study reports for the first time the presence of BRAF amplification in one KIT exon 11-mutated GIST (GIST_174). In the CNV analysis, patient GIST_174 also showed amplification of ETV1. Studies based on microRNAs strongly targeting ETV1 have showed that ETV1 inhibitors could have therapeutic potential in GIST management [36].

Regarding tumor suppressor genes, the deletion and the consequent loss of expression of CDKN2A/2B, located on the short arm of chromosome 9 at position 21.3, was observed in six patients in our study. The products encoded by this gene (p16INK4 and p14ARF) through the regulatory roles of CDK4 and p53, share a common functionality in cell cycle G1-phase control. Pan-CDK inhibitors such as flavopiridol (Alvocidib or HMR-1275) and palbociclib (PD0332991) are under investigation in many clinical trials. Moreover, loss of p16 is considered a prognostic factor for a high rate of malignancies and poor outcomes in patients with GISTs [37, 38]. Consistent with these findings, in our experiments no localized tumors showed deletion of this locus.

Loss of the upper arm of chr1 seemed to have an effect on the transcription level of CDKN2C, which encodes another cyclin-dependent kinase inhibitor (p18IN4) that controls cell cycle G1 progression through the interaction with CDK4 or CDK6. In particular, we found five cases with a significant down-modulation of CDKN2C expression. In the same chromosomal region, ARID1A monoallelic loss was detected in six of the seven samples. A broad spectrum of cancers has been shown to carry mutations in ARID1A, particularly deletion or nonsense mutations that are distributed throughout the ARID1A gene [39]. ARID1A is a member of the chromatin-remodeling SWI/SNF complex, which is involved in the regulation of many cellular processes, including differentiation, proliferation, DNA repair, and tumor suppression [40].

Notably, PTEN is the second most frequently mutated tumor suppressor gene in cancer after p53. This gene negatively regulates the PI3K/AKT/mTOR signaling pathway. Monoallelic loss with low protein expression has been shown to recur frequently in imatinib-resistant GIST, causing the activation of the PI3K/AKT/mTOR pathway [41, 42]. Moreover, this pathway has been shown to be activated in GIST with imatinib secondary resistance [43], representing a rational target for GIST therapy. To this end, several preclinical experiments and early studies have been conducted [44]. Some of them have showed limited success, probably due to the paradoxical reactivation of the PI3K pathway that occurs following mTORC1 inhibition. However, an upstream or more complete PI3K/AKT/mTOR pathway inhibition, or the association of inhibitors, such as everolimus and temsirolimus, with imatinib, may represent innovative therapeutic strategies to maximize blockade of the mTOR pathway and lead to greater antitumor activity. In our study, we showed that loss of this gene occurred in three of the seven patients with metastatic GIST while no patients with localized tumors showed deletion of this locus, suggesting a role of PTEN in the progression of this pathology. Additionally, we report for the first time in advanced GIST a nonsense mutation of PTEN in association with a loss of heterozygosity event that leads to depletion of mRNA expression.

Another interesting observation is the involvement of DMD gene in our patients (GIST_131, GIST_174, GIST_178 and GIST_188), as it has recently been reported in cancers with myogenic programs. DMD is the dystrophin gene, which is the largest known human gene and is located on the short arm of the X chromosome at position 21.2. Wang and colleagues identified DMD deletion as shared factor contributing to the development of myogenic cancers [45], hypothesizing that DMD inactivation promotes metastatic potential due to its role in regulating migration, invasion, anchorage and invadopodia formation. Recently our group has also reported the recurrence of dystrophin deletion in nine of 35 GIST samples [14]. These were all KIT/PDGFRα-mutant GISTs, whereas none of the 6 KIT/PDGFRα wild-type GIST samples showed DMD alterations. From a clinical point of view, patients harboring this alteration have more aggressive features and almost all have metastatic disease. Since dystrophin interacts with a complex of other proteins and glycoproteins, it is possible to compensate for dystrophin deficiency using them as potential point of therapeutic attack, as postulated in the development of new treatments for muscular dystrophies. As a result, treatment options under evaluation in clinical trials for Duchenne dystrophy also warrant evaluation for the treatment of GISTs and myogenic cancer.

SMARCB1, a core subunit of the SWI/SNF complex, is a known tumor suppressor and its loss was associated with rhabdoid tumor onset [46, 47]. In our work, four of the seven metastatic GISTs harbored a heterozygous deletion of part or the entire arm of chromosome 22, on which SMARCB1 is located, resulting in a significant down-regulation of SMARCB1 mRNA expression. Several pathways are regulated through the SMARCB1 and SWI/SNF complex, such as chromatin remodeling, cyclin D1/CDK4 activation, WNT/β-Catenin and sonic hedgehog signaling [48]. In particular, SMARCB1 was shown to interact with GLI molecules and, through its loss, the Hedgehog (Hh) pathway could be activated [49]. Activation of this pathway has been implicated in the development of various cancers and several molecules modulating its activity have been developed. The description of a GIST-like mouse model triggered by the inactivation of PTCH1 suggests that Hh could also have a role in GIST biology [50]. To our knowledge there are no published data evaluating the effects of Hedgehog inhibition in GIST. In our work, we tested the *in vitro* efficacy of GANT61 (a GLI-inhibitor) and demonstrated that this treatment could affect the viability of GIST cells and modulate the expression of key genes for tumor progression, such KIT and CDKN1A. These data are a first attempt to evaluate the effects of Hh inhibitors in GIST cell lines; further studies are needed to assess the consistency of these results in other GIST cell lines in association with different mutational backgrounds.

Our findings provide a more comprehensive molecular background for GISTs and improve our understanding of genomic aberrations and processes that drive GIST tumorigenesis, tumor progression, and malignancy.

These data, even though preliminary and not found in large series, may identify druggable targets for new treatments that could provide additional therapeutic options on a patient-specific basis. In particular, since almost all GISTs harboring KIT exon 11 mutations develop a resistance to TKIs, these data have important clinical implications that should be investigated further. Furthermore, using this approach, we detected and described aberrations that have not been previously reported in GIST, thereby contributing to a more detailed molecular understanding of this disease and identifying a possible point of departure for validating new prognostic markers associated with disease progression.

In conclusion, we found additional genomic alterations beyond those related to the ‘classic’ KIT receptor, even if they are not shared by all GIST samples. Further studies are needed in order to uphold our findings and to increase the dataset; additional supporting data may validate new genomic events that could be useful for clinical and molecular classification of GIST patients, identification of new targets, and the development of novel therapeutics.

## MATERIALS AND METHODS

This study on genome sequencing was approved by local Ethical Committee and the genomic analysis was done after the patients' written consent. This study was approved by the institutional review board of Azienda Ospedaliero-Universitaria Policlinico S. Orsola-Malpighi, Bologna, Italy (approval number 113/2008/U/Tess).

### Patient selection and tumor sample collection

Seven patients with metastatic GIST harboring a KIT exon 11 mutation were studied. The tumor and patient characteristics are illustrated in Table 1. All patients underwent surgery after treatment with imatinib or sunitinib. Tumor specimens of the primary lesions were collected, immediately frozen and stored until NGS study. For all metastatic samples, matched peripheral blood was collected during a follow-up visit. Additionally, tumor specimens of eight patients with localized KIT exon 11-mutant GIST were collected as control group for CN analysis.

### Next generation sequencing

Whole-transcriptome sequencing was performed on RNA isolated from fresh-frozen tumor tissue of seven patients with KIT exon 11-mutant metastatic GIST and whole exome sequencing was done on isolated DNA from matched peripheral blood.

Total RNA was isolated from fresh-frozen tumor tissues using the RNeasy spin-column method (Qiagen, Milan, Italy). Whole-transcriptome RNA libraries were prepared in accordance with Illumina's TruSeq RNA Sample Prep v2 protocol (Illumina, San Diego, California). Poly(A)-RNA molecules from 500 ng of total RNA were purified using oligo-dT magnetic beads. Following purification, the mRNA was fragmented and randomly primed for reverse transcription followed by second-strand synthesis to create double-stranded cDNA fragments. These cDNA fragments went through a terminal-end repair process and ligation using paired-end sequencing adapters. The products were then amplified to enrich for fragments carrying adapters ligated on both ends and to add additional sequences complementary to the oligonucleotides on the flow cell, thus creating the final cDNA library.

DNA was extracted from peripheral blood with QiaAmp DNA mini kit (Qiagen) following manufacturer's instructions. Whole exome libraries were prepared in accordance with Nextera Exome Enrichment protocol (Illumina). Briefly, 50 ng of genomic DNA was tagmented (tagged and fragmented) by the Nextera transposome technique to an average library size of 300-350 bp. The Nextera transposome simultaneously fragments the genomic DNA and adds adapter sequences to the ends. DNA libraries were denatured to single stranded DNA and hybridized to biotin-labeled 95-mer probes designed to enrich more than 200000 exons, spanning 20794 genes, including exon-flanking regions, then eluted from magnetic beads.

RNA and DNA library size were checked and sized with Agilent DNA 1000 chips on the Bioanalyzer 2100 (Agilent Technologies, Taiwan), then libraries were quantified using both PicoGreen assay (Life Technologies) and KAPA library quantification kit (KAPA Biosystem, Boston, USA). 12pM paired-end libraries were amplified and ligated to the flowcell by bridge PCR, and sequenced at 2×75bp read length for RNA and 2×100bp for exome sequencing, using Illumina Sequencing by synthesis (SBS) technology. An average of 77 and 34 million reads per sample were obtained for WTS and WES analysis respectively.

### Bioinformatic analysis

The short reads were processed and mapped on the human reference genome by TopHat/Bowtie pipeline, while variation calling was performed with SAMtools and SNVMix2, thus identifying all the point mutations, insertions and deletion present in the sample (SNV and InDels). Variants present in dbSNP and 1000 Genomes with frequency greater than 1% were excluded. All variants from the matched normal-tumor pairs that were unique in the tumor sample were called as somatic. The effect of coding SNV was predicted at the protein level with a suite of computational tools, such as SNPs&GO and PROVEAN. Truncations and frameshift mutations were analyzed in relation to the annotations available on the protein sequence (eg from UniProt, PFAM, SCOP) in order to identify possible domain/site loss, disruption or gain that can affect protein function. Large chromosomal rearrangements were detected with several bioinformatic tools (DeFuse, ChimeraScan and FusionMap).

### Gene expression analysis

RNA seq data were analyzed in order to evaluate the gene expression profile of the seven GIST samples. After the alignment procedure, the BAM file obtained was manipulated with SAMtools in order to remove the optical/PCR duplicate, and to sort and index it. The function HTSeq-count (Python package HTSeq) was adopted to count the number of reads mapped (cpm) on known genes, included in the Ensembl release 72 annotation features (http://www.ensembl.org).

### Copy number analysis

Genomic DNA extracted from eight localized and seven metastatic GIST specimens was labelled and hybridized to SNP array Genome Wide SNP 6.0 (Affymetrix) following manufacturer's instructions. Quality control was performed by Contrast QC and MAPD calculation. Copy number analysis was performed by Genotyping Console and visualized with Chromosome Analysis Suite (ChAS) Software (Affymetrix). Hidden Markov Model algorithm was used to detect amplified and deleted segments with stringent parameters. To control for hyperfragmentation, adjacent segments separated by < 50 probes were combined into one single segment, and only segments > 50 probes were considered.

### RT-PCR validation of fusion transcripts

RNA was reverse transcribed to cDNA using the Transcriptor First-Strand cDNA Synthesis Kit (Life Technologies) with oligo dT primers. Primers specific for the breakpoint flanking regions of the fusion transcripts, identified with RNA-seq analysis, were used to amplify and sequence the fusion transcript using the Sanger method.

### Cell line culture and treatment

GIST882 (kindly provided by Dr. Jonathan A. Fletcher, Harvard Medical School, Boston, Massachusetts, USA) was cultured in RPMI1640+15% FBS. The GIST882 cell line is characterized by monosomy of chromosome 22 [51] and thus was used as a GIST model of SMARCB1 monoallelic loss. The GLI inhibitor GANT61 (Calbiochem, San Diego, CA) was used to evaluate the effects, on mRNA and viability, of Hedgehog inhibition. Cells were cultured for 72 hrs in a medium supplemented with GANT61 (concentrations range 6.25-100 μM). To assess specificity, vehicle control samples (treated with 1.6% dimethyl sulfoxide [DMSO]) were added in each experiment.

### qPCR

For mRNA extraction, cells were seeded into a 24-well plate at a density of 3×10^5^ cells/well. After 72 hrs of treatment with GANT61, total RNA was extracted with RNA mini kit (Qiagen) and retro-transcription was performed with oligo-dT primers. qPCR amplification of the gene of interest was performed with real-time LightCycler 480 instrument (Roche). Fold-change was estimated by DDCt method, using ATPS and YWHAZ genes as housekeeping controls. The p value of differential expression with respect to vehicle control (DMSO-only) was estimated with a t test.

### Cell viability

For the cell viability measurement, cells were seeded in triplicate into a 96-well plate at a density of 4×10^4^ cells/well. After 72 hrs of treatment with GANT61, the vitality of the cells was assessed using by WST-1 assay (Roche) and percent of inhibition was evaluated with respect to vehicle control (DMSO only). A dose response curve was drawn for estimation of the inhibition of cell viability. GraphPad PRISM Software was used for graph design and statistical analysis.

## SUPPLEMENTARY MATERIAL FIGURES AND TABLE




