# Supplementary material for: Molecular characterization of metastatic exon 11 mutant gastrointestinal stromal tumors (GIST) beyond KIT/PDGFRα genotype evaluated by next generation sequencing (NGS)
Source: Oncotarget. 2015 Nov 2;6(39):42243–57. doi: 10.18632/oncotarget.6278 (PMC4747222; doi:10.18632/oncotarget.6278)
Supplement: Supplementary file 1 [file oncotarget-06-42243-s001.pdf]

Molecular characterization of metastatic exon 11 mutant gastrointestinal stromal tumors (GIST) beyond KIT/PDGFRα genotype evaluated by next generation sequencing (NGS)

Supplementary Material

A.

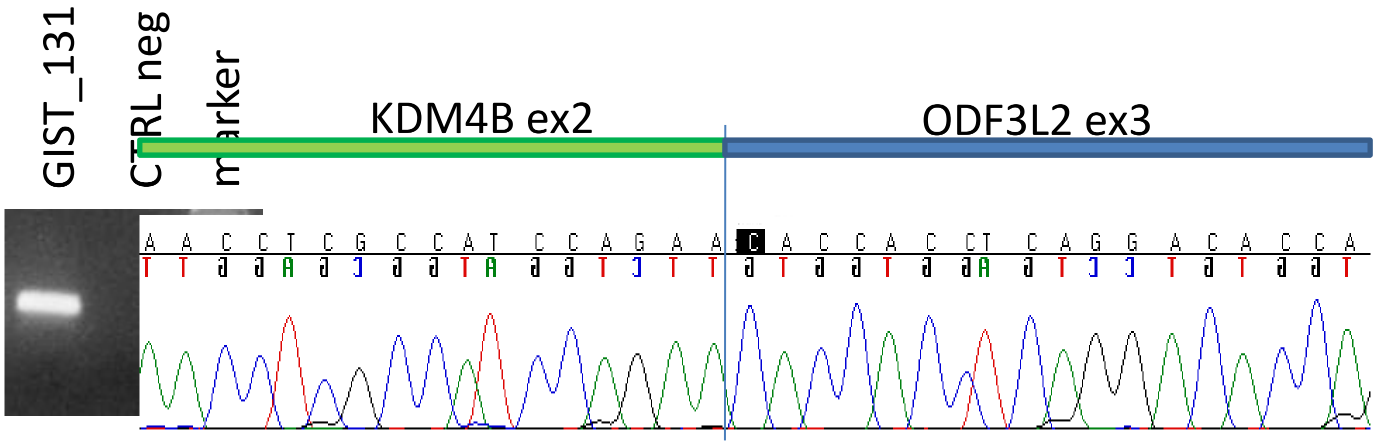

KDM4B-ODF3L2 fusion transcript detected in GIST_131

B.

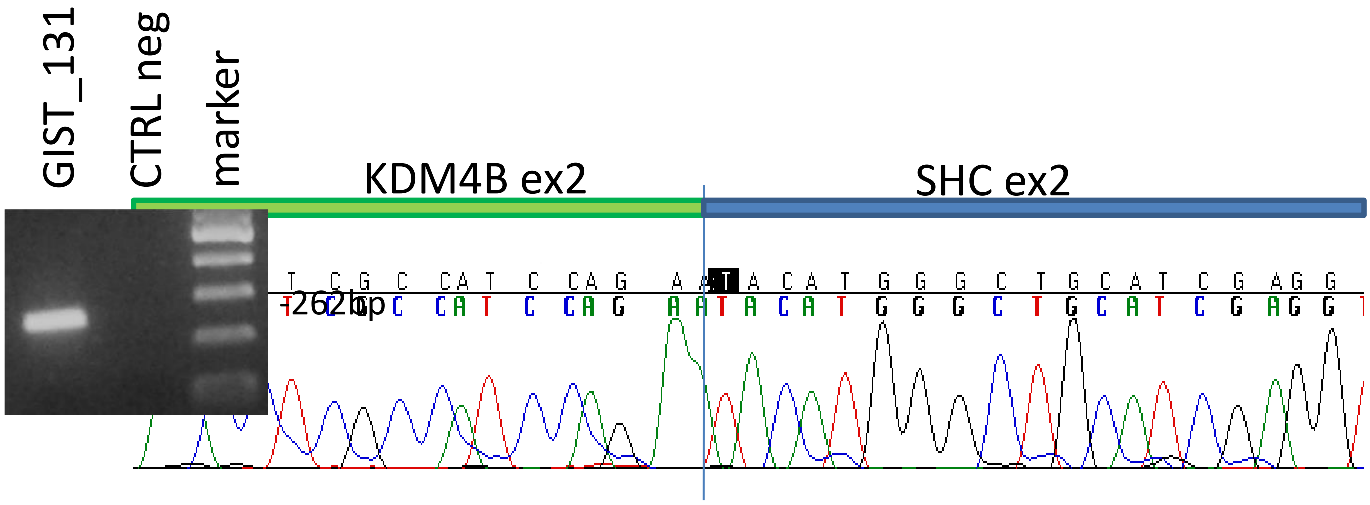

KDM4B-SHC2 fusion transcript detected in GIST_131

C.

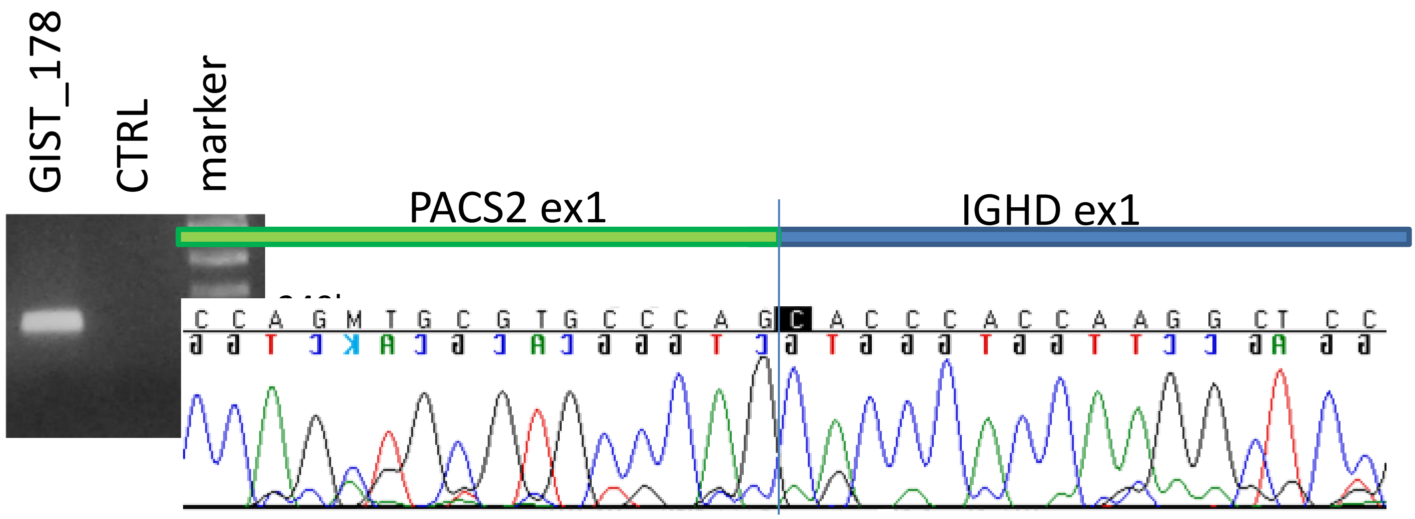

PACS2-IGHD fusion transcript detected in GIST_178

D.

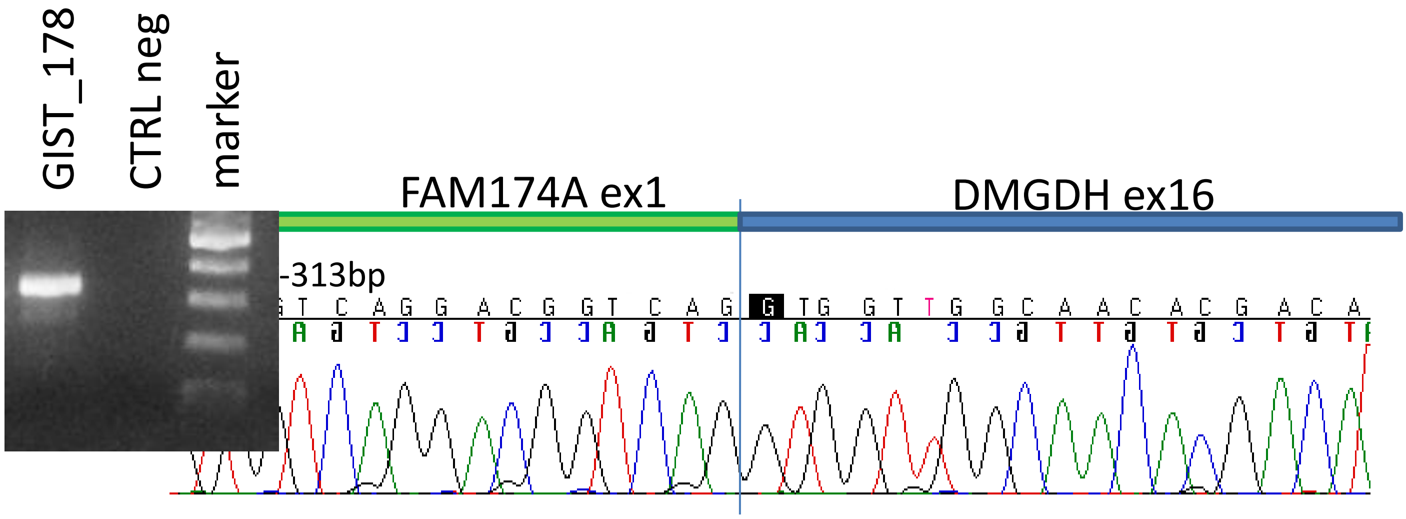

FAM174A-DMGDH fusion transcript detected in GIST_178

E.

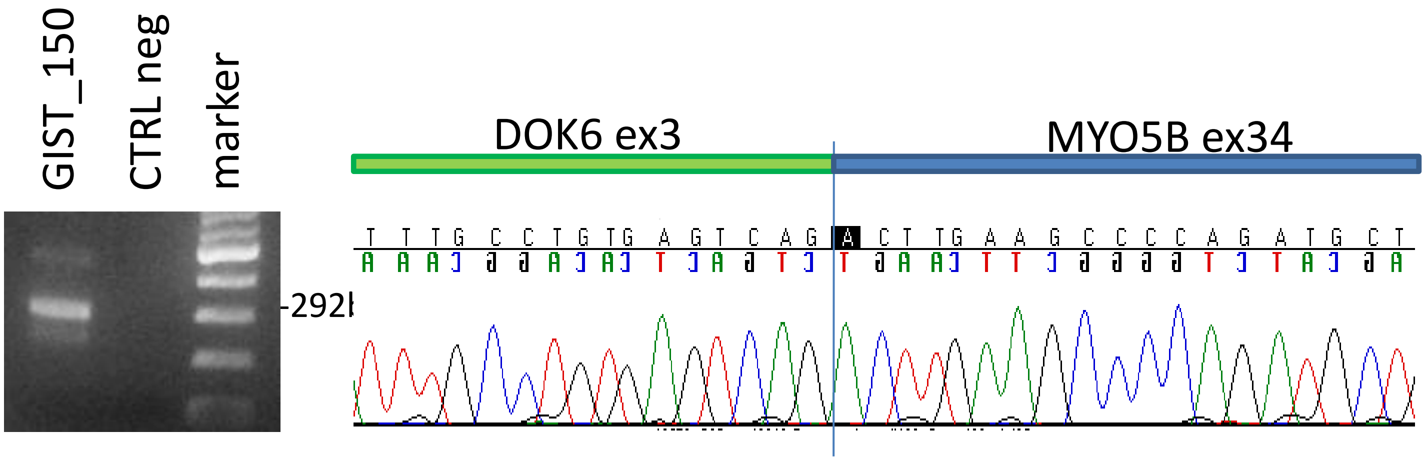

DOK6-MYO5B fusion transcript detected in GIST_150

F.

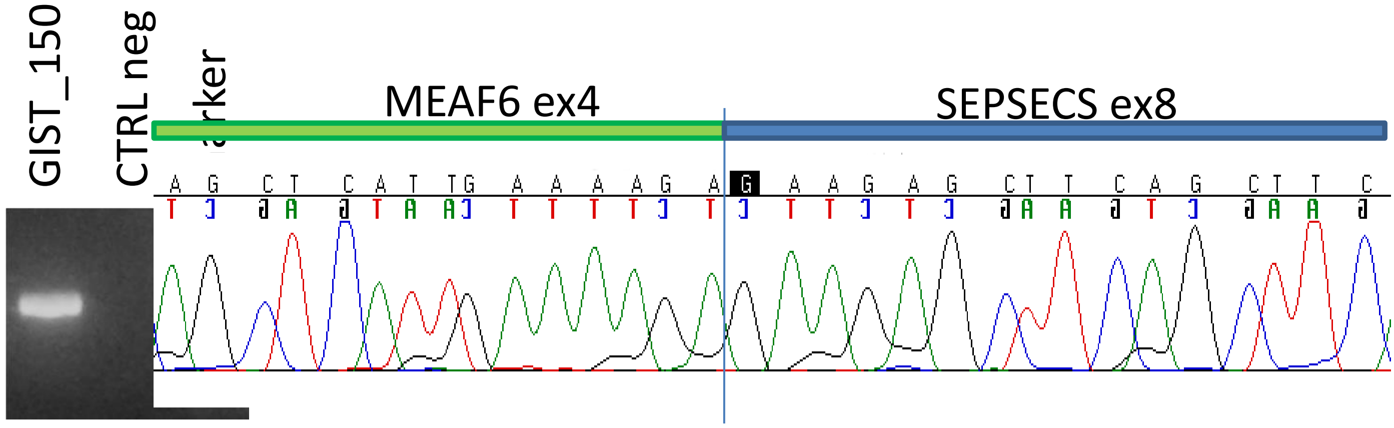

MEAF6-SEPSECS fusion transcript detected in GIST_150

Figure S1. Validation with RT-PCR and Sanger Sequencing of the seven fusion transcripts detected by RNA-sequencing.

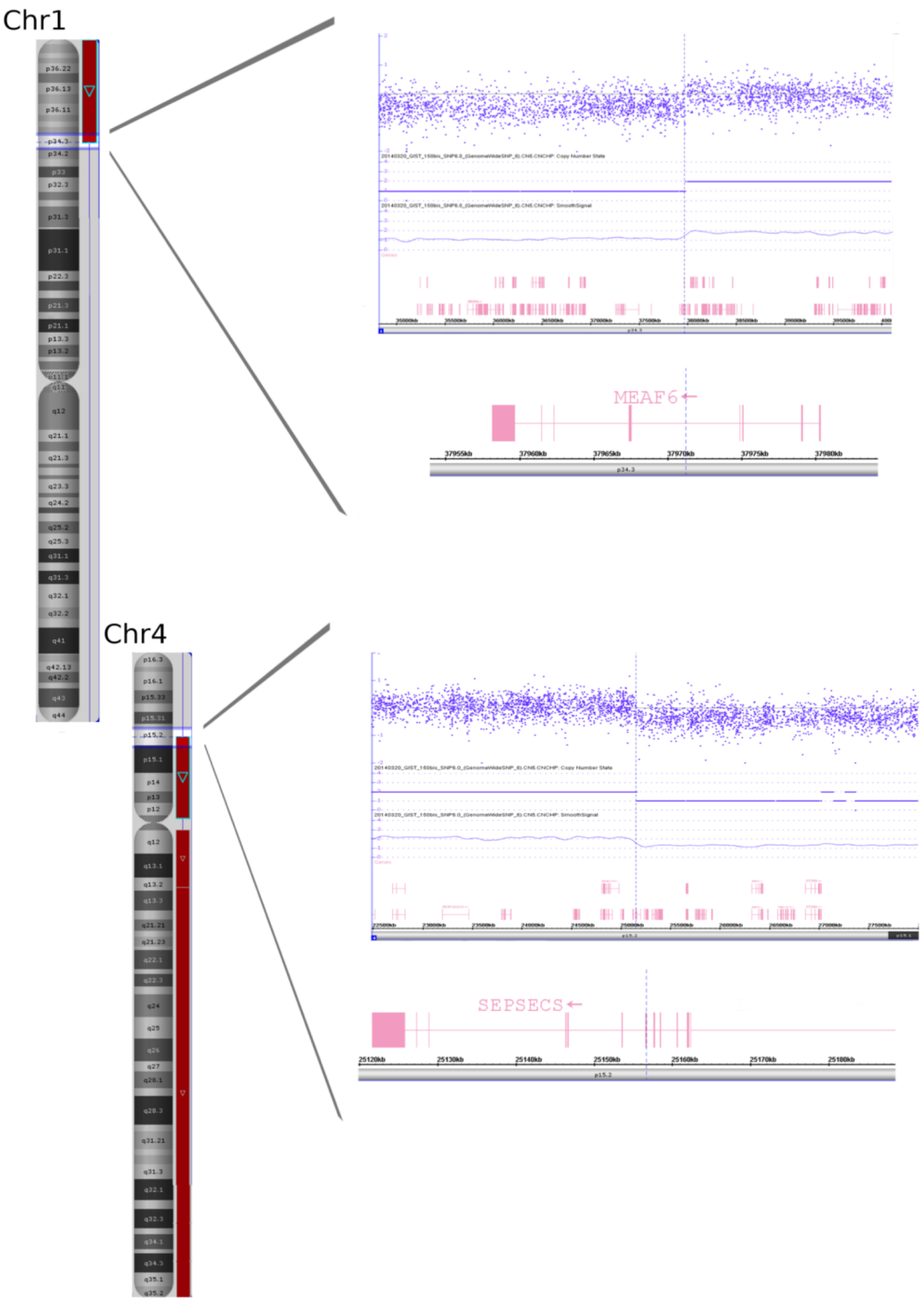

Figure S2.

Copy number variation analysis performed on chromosome 1 and 4 of GIST_150. The loss of the region between p36.22 and p34.3 of chromosome 1 and the loss of the region between p15.2 and q35.2 of chromosome 4 indicated the presence of the unbalanced translocation t(1;4)(p34.3;p15.2). This translocation was supported by the detection of MEAF6-SEPSECS chimeric transcript with RNA-seq (showed

in supplementary figure 1F). Both genes are encoded from the minus strand and are located in the breakpoint regions of this translocation.

Table S1. Copy number variations and mutational landscape of the most significant genes, detected by SNP-array and WTS respectively.
